# Application of functional genomics for domestication of novel non-model microbes

**DOI:** 10.1093/jimb/kuae022

**Published:** 2024-06-26

**Authors:** Margaret K Bales, Michael Melesse Vergara, Carrie A Eckert

**Affiliations:** Biosciences Division, Oak Ridge National Laboratory, Oak Ridge, TN 37831, USA; Bredesen Center for Interdisciplinary Research, Graduate School of Genome Science and Technology, University of Tennessee, Knoxville, TN 37996, USA; Center for Bioenergy Innovation, Oak Ridge National Laboratory, Oak Ridge, TN 37831, USA; Biosciences Division, Oak Ridge National Laboratory, Oak Ridge, TN 37831, USA; Center for Bioenergy Innovation, Oak Ridge National Laboratory, Oak Ridge, TN 37831, USA; Biosciences Division, Oak Ridge National Laboratory, Oak Ridge, TN 37831, USA; Bredesen Center for Interdisciplinary Research, Graduate School of Genome Science and Technology, University of Tennessee, Knoxville, TN 37996, USA; Center for Bioenergy Innovation, Oak Ridge National Laboratory, Oak Ridge, TN 37831, USA

**Keywords:** Genome-scale screening, Synthetic biology, CRISPRi

## Abstract

With the expansion of domesticated microbes producing biomaterials and chemicals to support a growing circular bioeconomy, the variety of waste and sustainable substrates that can support microbial growth and production will also continue to expand. The diversity of these microbes also requires a range of compatible genetic tools to engineer improved robustness and economic viability. As we still do not fully understand the function of many genes in even highly studied model microbes, engineering improved microbial performance requires introducing genome-scale genetic modifications followed by screening or selecting mutants that enhance growth under prohibitive conditions encountered during production. These approaches include adaptive laboratory evolution, random or directed mutagenesis, transposon-mediated gene disruption, or CRISPR interference (CRISPRi). Although any of these approaches may be applicable for identifying engineering targets, here we focus on using CRISPRi to reduce the time required to engineer more robust microbes for industrial applications.

**One-Sentence Summary:**

The development of genome scale CRISPR-based libraries in new microbes enables discovery of genetic factors linked to desired traits for engineering more robust microbial systems.

## Graphical Abstract

**Graphical Abstract:**
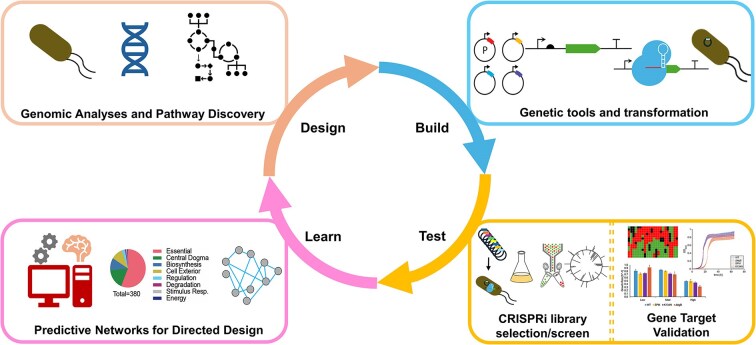

## Introduction

**Fig. 1.**
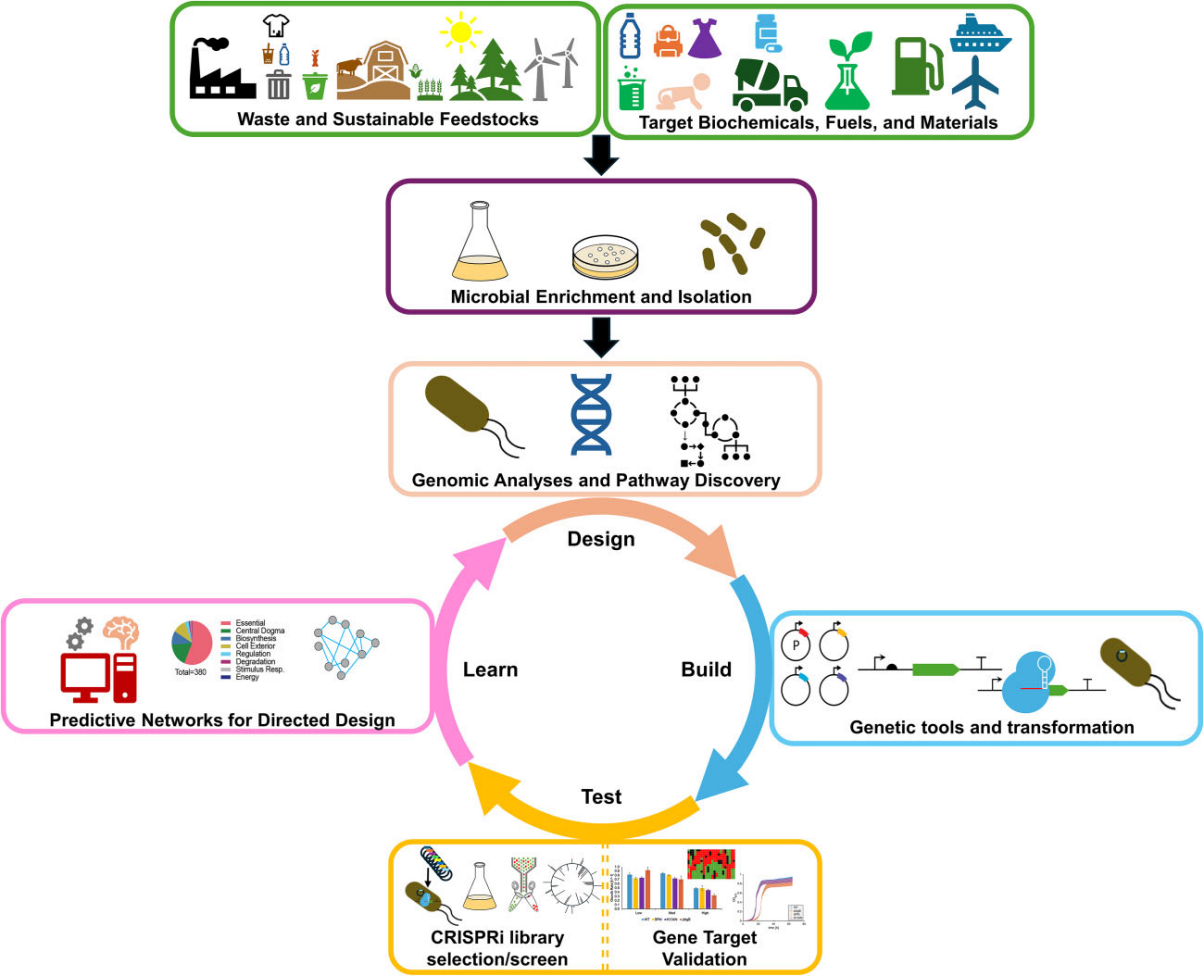
Design-Build-Test-Learn (DBTL) tools toward a circular bioeconomy. Supporting a circular bioeconomy requires the utilization of a variety of unconventional feedstocks including waste streams and renewable feedstocks such as woody biomass. Environmental microbes provide a largely untapped source of enzymes and pathways towards utilization of these feedstocks and production of a wide variety of biochemicals and materials. Domesticating these microbes for industrialization requires effective characterization including genome sequencing and –omics analysis. With this information, genetic tools can be developed and applied towards engineering and implementation of functional genomic screens and selections to identify genotype-phenotype relationships to guide further engineering. This involves a DBTL strategy to inform the most efficient routes to domestication and industrialization and provides data for better predictive design. This increase in capabilities accelerates efforts towards sustainable industrial practices and generally improves understanding of microbial diversity.

As consumers realize the environmental and social effects of unsustainable industrial practices and change their purchasing behavior, it will be critical for all industries to explore and invest in sustainable practices (Bradu et al., 2023). The use of biological processes to alter industry away from the linear consumption of resources to one where reusability is a priority, defines the emerging circular bioeconomy (Hadley Kershaw et al., 2021). Microbes are critical for the development of a sustainable and resilient bioeconomy (Ma et al., 2022). In selecting a microbe for a particular industrial process, it is vital to ensure a base level of tolerance to conditions under which it will be applied, reducing the risk associated with attempting to adapt a model microorganism to harsh and inhospitable growth conditions such as low pH or high temperature (Liu et al., 2022). Transferring metabolic pathways of interest from undomesticated microbes into easy-to-manipulate hosts is also likely to introduce the need for adaptation due to redox imbalances or pathway intermediate/product toxicity. Here, we will discuss our perspective on the associated challenges with domesticating new microbes and the steps required to develop an unbiased, genome-scale, functional screen or selection that allows for efficient and rapid genotype-phenotype discovery in a microbe targeted for industrial applications (Fig. 1). We will also discuss the emerging need and potential for developing genetic tools applicable to a broader range of microbes, including extremophilic organisms.

## Discussion

### Microbial Isolate Characterization and Genetic Tool Development

Isolating microbes from conditions matching their intended industrial use environment, that is utilization of waste feedstocks, high temperature, high salt, or extreme pH, will ensure microbes have the necessary features for optimal growth and production to reduce time to economic viability. Exploring more extreme environments can also reveal metabolomic mechanisms that can be more easily adopted for industrial applications (Beloqui et al., 2008). The sequencing cost of third generation long-read technologies has continued to reduce and the quality and depth of data is sufficient for *de novo* genome and metagenome assemblies (Athanasopoulou et al., 2022). Combining long-read based genome assemblies with short-read polishing achieves high genome sequencing accuracy (Zhang et al., 2021) and reveals critical information about the accessible metabolic output present in newly isolated microbes. Initial metabolomic and proteomic surveys can additionally provide substantial functional information that is difficult to assess through genome sequencing alone (Bauermeister et al., 2022; Bludau & Aebersold, 2020).

Genetic manipulation of isolated microbes is indispensable for improving function in industrial applications (Becker & Wittmann, 2018). One barrier to genomic engineering efforts is the innate restriction-modification (R-M) systems found in most bacteria and archaea. Methylome sequencing using PacBio or ONT helps characterize DNA methyltransferases (MTases) that are active in each host (Crits-Christoph et al., 2023; Li et al., 2020; Tourancheau et al., 2021) and accounting for the methylation state of the host can provide immediate increases in transformation efficiency (Riley & Guss, 2021). Establishing functional genetic tools is the next critical step. These include, but are not limited to, compatible origins of replication, promoters, ribosome binding sites, and terminators to enable controlled maintenance and expression of genetic components (Freed et al., 2018). Chromosomal integration is also generally practical as replicating plasmids can be unstable and vary in copy number in host microbes. Integrated DNA is more stable in the host and reduces the need for antibiotic-based selection. Serine integrase and recombinase-based systems can remove host range limitations of a replicating plasmid and the efficiency of native homologous recombination to evaluate heterologous genes and pathways (Ba et al., 2023; Elmore et al., 2023).

### Genome-scale Screening and Selection Methods

Genome-scale studies are an essential step in developing a host for industrial uses as they enable the identification of genetic factors associated with complex traits for strain optimization. There are a variety of methods that have been employed including adaptive laboratory evolution (Dragosits & Mattanovich, 2013; Lim et al., 2021; Mueller et al., 2022; Wang et al., 2023; Zhan et al., 2023), transposon-mediated gene disruption (Tn-Seq) (Bleem et al., 2023; Borchert et al., 2023; Price et al., 2018; Shields & Jensen, 2019; Wetmore et al., 2015), CRISPR-based modifications including interference (CRISPRi), CRISPR activation (CRISPRa), and base editing (Adli, 2018; Lee et al., 2019; Li et al., 2016; Liu et al., 2022; Saber Sichani et al., 2023; Silvis et al., 2021; Volke et al., 2023), and random mutagenesis (Freed et al., 2018; Riley & Guss, 2021). Herein we will focus on CRISPRi, a recently emerged robust functional genomic screening and selection method (Trivedi et al., 2023). CRISPRi is a gene repression technique that utilizes a catalytically inactive Cas9 nuclease (dCas9) complexed with a single-guide RNA (sgRNA), expressed from single gRNA bearing plasmids, that directs it to a targeted region in the genome, binds the DNA, and prevents transcription (Call & Andrews, 2022; Cho et al., 2018; de Bakker et al., 2022; Schultenkämper et al., 2020; Todor et al., 2021; Zhang et al., 2021). Inducible expression of dCas9 provides unique abilities not possible with other techniques such as TN-Seq, including probing essential genes and seeing a range in phenotypes resulting from varying levels of repression within the same gene targeted by multiple sgRNAs (Cui et al., 2018; Li et al., 2020; Rousset et al., 2018; Wang et al., 2018).

As with any experimental tool, there are limitations to dCas9 use that must be considered. One of the most frequently encountered issues is toxicity of dCas9 protein expression (Vento et al., 2019). Placing dCas9 under an inducible, titratable promoter is one strategy to alleviate this issue. Cas9 requires a three base pair (NGG) PAM (protospacer adjacent motif) that directly impacts the number of genomic sites available for binding, potentially limiting the number of gRNAs that can be targeted towards a particular gene (Collias & Beisel, 2021). Large amounts of engineering work to expand and alter PAM specifications has been done to address this limitation (Leenay & Beisel, 2017). Additionally, there is the potential for off-target knockdown effects plus a lack of generalizable gRNA design rules between species (Cui et al., 2018; Tadić et al., 2019). A few library design rules are vital in constructing a comprehensive and targeted genome-scale library. Excluding sgRNAs with overlapping seed region sequences helps prevent off-target effects and including enough sgRNAs (∼10) per gene in the library will ensure effective gene repression by at least one sgRNA, as *a priori* prediction of repression strength for individual sgRNAs is difficult. Additionally, multiple sgRNAs targeting each gene will reveal phenotypes resulting from varying levels of gene repression that might be missed by gene knockout or only one level of repression. As the available CRISPRi functional genomics datasets increase, refinements to general gRNA design rules, like those demonstrated for *E. coli* (Noshay et al., 2023; Yu et al., 2024), will improve CRISPRi experimental design for non-model microbes. Further, as more nucleases such as Cas12a are developed for genomic engineering applications (Ma et al., 2022), these proteins are being adopted as useful alternatives, should dCas9 expression toxicity pose an issue in a given system. The ability to multiplex gRNAs in a single array and utilize Cas12a in low-GC content non-model organisms has broadened the applicability of Cas-based functional genomic screens (Choi & Woo, 2020; Fleck & Grundner, 2021; Jervis et al., 2021; Joseph & Sandoval, 2023; Schilling et al., 2020). The development of other nucleases, such as Cas12a, will expand the knowledge gained from Cas-based transcriptional interference studies, thus bolstering the available data for metabolic engineering applications.

Sufficient transformation efficiency for adequate library coverage is also an important consideration when implementing CRISPRi libraries. For organisms with poor transformation efficiency, the size of an effectively transformable library will be limited. To achieve successful target suppression and realize the benefits of partial gene knockdowns, selecting fewer genes and providing sufficient sgRNA coverage across the target genes may be advantageous. Further, it is critical to make roughly ∼1% of the library non-targeting guides as an internal control for dCas9 toxicity. Well-designed CRISPRi libraries have been employed to study an ever-increasing number of biological phenomena, including improving growth on a desirable substrate, increasing product production, developing microbes as biosensors in the soil, and microfluidics for bioanalysis (Table 1).

**Table 1.** Examples of CRISPRi Studies.

Category	Application	Citation
Biosensors	1. Metabolic flux control 2. Compound over-production 3. Pollution detection 4. Product formation detection 5. Compound tolerance analysis	1. Chiang & Hasty (2023), Wu et al. (2020) 2. Peng et al. (2024), Shen et al. (2022), Wang et al. (2023) 3. Roy et al. (2021) 4. Henke et al. (2022) 5. Mormino et al. (2022)
Microfluidics	1. High-titer protein production 2. Antibiotic resistance determination 3. Enzyme secretion 4. Increased compound production 5. Synthetic circuits 6. Study large-scale genotypic variation	1. Li et al. (2016), Yu et al. (2023) 2. Kong et al. (2019) 3. Andreas Johansson et al. (2023) 4. Li et al. (2016) 5. Santos-Moreno et al. (2020) 6. Camsund et al. (2020)
Metabolic engineering	1. Enhanced fatty alcohol production 2. Single-cell protein production 3. Alter metabolic flux/pathway engineering 4. Amino acid production 5. Terpenoid synthesis 6. Growth-product coupling and decoupling 7. Commodity chemical production	1. Kaczmarzyk et al. (2018) 2. Balagurunathan et al. (2022) 3. Cho et al. (2018), Fenster et al. (2022), Lu et al. (2022), Xia et al. (2019), Zhao et al. (2021) 4. Yin et al. (2024) 5. Chu (2022) 6. Banerjee et al. (2020), Li et al. (2020) 7. Mougiakos et al. (2018)

### CRISPRi Screening and Selection

Experimental design is critical when setting up a pooled library selection or screen such as CRISPRi to ensure that unintended bias does not skew output data, leading to false positives or negatives. Therefore, carefully defining screen or selection conditions is one of the first considerations. For example, utilizing a carbon source without a defined composition will make assigning specific causes for observed phenotypes more challenging. Choosing a relatively straightforward or quantifiable phenotype such as survival, growth rate, or product output increases the interpretability of the selection or screen outcome. One can assess the effectiveness of the screen or selection by monitoring ‘known’ candidate hits with expected phenotypes (e.g. efflux pumps under antibiotic stress) (Kim et al., 2017). Initial analysis of CRISPRi screens or selections provides genome-wide expression knockdown to identify genotype-to-phenotype relationships more clearly. Genes of interest are identified by assessing the log_2_ fold change of the final abundance over the initial abundance for each sgRNA in the library as normalized by internal non-targeting gRNA controls and control library growth conditions. These data uncover specific gene knockdown enrichments or depletions reflecting changes in growth and survival under experimental conditions (Bock et al., 2022). Once these data are compiled and evaluated, subsequent validation of gene targets can be chosen to recapitulate the observed phenotype. These validation experiments may include individual and/or combinatorial gene knockdowns, promoter substitutions, or complete gene deletions and subsequent phenotypic evaluations such as proteomics, activity assays, and microscopy to identify modes for improved growth under the chosen growth conditions (Anglada-Girotto et al., 2022; Liu et al., 2017; Wang et al., 2023; Zhan et al., 2020).

Effective application of CRISPRi functional genomic analyses is dependent on well-developed genetic tools in the organism of interest (Yeom et al., 2023). Excellent transformation efficiency, well characterized plasmid components (origins of replication, selectable markers, and promoters) and genomic integration tools are critical for implementing well-designed experiments. As novel, extremophilic organisms, with few of the necessary genetic tools are adopted for industrial applications, it is essential to invest effort in developing the appropriate genetic toolkits.

### Future Applications: Expanding Microbial Chassis

A growing number of industrially relevant microbes are emerging as production chassis using various carbon sources (Fatma et al., 2020; Heijstra et al., 2017; Nargotra et al., 2023). Industrial microbes will increasingly be isolated from extreme growth conditions to optimize industrial processes and decrease costs. Advantages of developing extremophiles for industrial applications include native production of thermostable enzymes with higher substrate solubility, higher conversion efficiency, increased reaction kinetics, and decreased risk of contamination (Zhu et al., 2020). Additionally, some feedstock substrates of interest, like polyethylene terephthalate, are not amenable to biological valorization at mesophilic temperatures (Orlando et al., 2023). Developing thermophilic microbes able to metabolize such substrates have the potential for improving resource recycling. Realizing the industrial potential of these microbes will require rapid development of amenable genetic tools. As it currently stands, there is a lack of broadly applicable genetic tools, including selectable markers, promoters, plasmid origins, and transformation protocols, as broadly utilized mesophilic tools are not functional in these thermophilic microbes (Y. Wang et al., 2024; Ye et al., 2023). While there are a growing number of tools being generated for use in thermophilic microbes (Adalsteinsson et al., 2021; Le & Sun, 2022; Riley et al., 2019; Walker et al., 2020; Wang et al., 2022; Wu et al., 2023; Yang et al., 2023), developing new tools and implementing CRISPR mediated genome engineering and/or thermostable serine recombinase-assisted genome engineering (SAGE) mediated integration would greatly simplify testing production of various industrial chemicals in these currently underutilized microbes (Fenster & Eckert, 2021; Wu et al., 2023). Continued decrease in -omics costs coupled with advanced genome modification techniques makes adopting extremophiles and other novel, less studied microbes as industrial chassis a viable and exciting avenue of research that should be more intensely pursued.
